# Tetra­aqua­bis­{2-[4-(4-pyrid­yl)pyrimidin-2-ylsulfan­yl]acetato}­zinc

**DOI:** 10.1107/S160053681102993X

**Published:** 2011-07-30

**Authors:** Hai-Bin Zhu, Xin Lu

**Affiliations:** a School of Chemistry and Chemical Engineering, Southeast University, Nanjing 211189, People’s Republic of China

## Abstract

In the title compound, [Zn(C_11_H_8_N_3_O_2_S)_2_(H_2_O)_4_], the Zn^II^ ion lies on an inversion centre and is coordinated by four water mol­ecules and two N atoms from two 2-[4-(4-pyrid­yl)pyrimidin-2-ylsulfan­yl]acetate (*L*) ligands in a distorted octa­hedral geometry. In *L*, the pyridine and pyrimidine rings are twisted at an angle of 11.2 (1)°. The coordinated water mol­ecules and the acetate groups are involved in the formation of a three-dimensional hydrogen-bonded network, which consolidates the crystal packing.

## Related literature

For a related structure, see: Zhu *et al.* (2009 ▶).
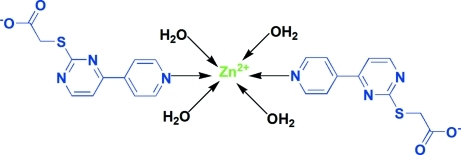


## Experimental

### 

#### Crystal data

[Zn(C_11_H_8_N_3_O_2_S)_2_(H_2_O)_4_]
*M* *_r_* = 630.00
Orthorhombic, 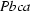

*a* = 7.199 (7) Å
*b* = 11.792 (11) Å
*c* = 28.77 (3) Å
*V* = 2442 (4) Å^3^
*Z* = 4
Mo *K*α radiation
μ = 1.24 mm^−1^
*T* = 298 K
0.20 × 0.20 × 0.15 mm

#### Data collection

Bruker APEXII CCD area-detector diffractometer
Absorption correction: multi-scan (*SADABS*; Bruker, 2001 ▶) *T*_min_ = 0.780, *T*_max_ = 0.830
16776 measured reflections
2465 independent reflections
1836 reflections with *I* > 2σ(*I*)
*R*_int_ = 0.051

#### Refinement

*R*[*F* ^2^ > 2σ(*F* ^2^)] = 0.033
*wR*(*F* ^2^) = 0.086
*S* = 1.02
2465 reflections
190 parameters
4 restraints
H atoms treated by a mixture of independent and constrained refinement
Δρ_max_ = 0.23 e Å^−3^
Δρ_min_ = −0.45 e Å^−3^

### 

Data collection: *APEX2* (Bruker, 2007 ▶); cell refinement: *SAINT-Plus* (Bruker, 2007 ▶); data reduction: *SAINT-Plus*; program(s) used to solve structure: *SHELXS97* (Sheldrick, 2008 ▶); program(s) used to refine structure: *SHELXL97* (Sheldrick, 2008 ▶); molecular graphics: *SHELXTL* (Sheldrick, 2008 ▶); software used to prepare material for publication: *SHELXTL*.

## Supplementary Material

Crystal structure: contains datablock(s) I, global. DOI: 10.1107/S160053681102993X/cv5136sup1.cif

Structure factors: contains datablock(s) I. DOI: 10.1107/S160053681102993X/cv5136Isup2.hkl

Additional supplementary materials:  crystallographic information; 3D view; checkCIF report

## Figures and Tables

**Table 1:** Hydrogen-bond geometry (Å, °)

*D*—H⋯*A*	*D*—H	H⋯*A*	*D*⋯*A*	*D*—H⋯*A*
O4—H4*WB*⋯O2^i^	0.81 (2)	1.96 (2)	2.759 (3)	170 (3)
O4—H4*WA*⋯O1^ii^	0.82 (2)	1.82 (2)	2.632 (3)	172 (2)
O3—H3*WB*⋯O2^iii^	0.84 (2)	1.89 (2)	2.728 (3)	176 (3)
O3—H3*WA*⋯O2^iv^	0.82 (2)	2.24 (2)	3.060 (3)	172 (3)

Symmetry codes: (i) 
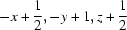
; (ii) 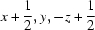; (iii) 
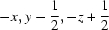
; (iv) 
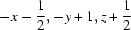
.

**Fig. 1.**
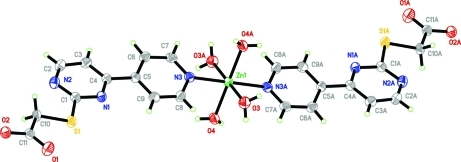
The coordination environment around Zn(II) in the title complex with the atom-labeling scheme [symmetry code: (A) -x, -y + 1, -z + 1]. Displacement ellipsoids for non-hydrogen atoms are drawn at the 50% probability level.

## supplementary crystallographic information

### Comment

In our previous work, we have reported the crystal structure of mononuclear Mn(II) complex with the ligand of 2-(4-(pyridine-3-yl)pyrimidin-2-ylthio)acetic acid (Zhu *et al.*, 2009). Herein, we present a Zn(II) complex with the ligand of 2-(4-(pyridine-4-yl)pyrimidin-2-ylthio)acetic acid.

Similar to the reported Mn(II) coordination compound (Zhu *et al.*, 2009), the Zn(II) center in the title compound also adopts an octahedral coordination geometry defined by four water O atoms in equatorial positions and two N atoms in apical positions (Fig. 1). The Zn—O bond lengths vary from 2.070 (2) to 2.137 (2)Å, and the Zn—N bond length is 2.176 (2) Å. Intermolecular O—H···O hydrogen bonds (Table 1) consolidate the crystal packing.

### Experimental

The mixture of Zn(NO_3_)_2_ (0.1 mmol), *L* (0.2 mmol) and NaOH (0.2 mmol) in 10 ml of H_2_O was stirred for 30 min at room temperature. After filtration, the mother liquid was stood for three weeks to give yellow crystals suitable for X-ray diffraction analysis.

### Refinement

C-bound H atoms were positioned geometrically (C—H = 0.93 Å) and allowed to ride on their parent atoms, with *U*_iso_(H) = 1.2 *U*_eq_(C). The positions of the water H atoms were found from a difference Fourier map and refined with restraint O—H = 0.82 (2) Å using a riding model, with *U*_iso_(H) = 1.2 *U*_eq_(O).

### Crystal data

**Table:**

[Zn(C_11_H_8_N_3_O_2_S)_2_(H_2_O)_4_]	*F*(000) = 1296
*M**_r_* = 630.00	*D*_x_ = 1.714 Mg m^−^^3^
Orthorhombic, *P**b**c**a*	Mo *K*α radiation, λ = 0.71073 Å
Hall symbol: -P 2ac 2ab	Cell parameters from 2465 reflections
*a* = 7.199 (7) Å	θ = 2.3–25.5°
*b* = 11.792 (11) Å	µ = 1.24 mm^−^^1^
*c* = 28.77 (3) Å	*T* = 298 K
*V* = 2442 (4) Å^3^	Block, yellow
*Z* = 4	0.20 × 0.20 × 0.15 mm

### Data collection

**Table:**

Bruker APEXII CCD area-detector diffractometer	2465 independent reflections
Radiation source: fine-focus sealed tube	1836 reflections with *I* > 2σ(*I*)
graphite	*R*_int_ = 0.051
φ and ω scans	θ_max_ = 27.1°, θ_min_ = 1.4°
Absorption correction: multi-scan (*SADABS*; Bruker, 2001)	*h* = −8→9
*T*_min_ = 0.780, *T*_max_ = 0.830	*k* = −14→14
16776 measured reflections	*l* = −34→32

### Refinement

**Table:**

Refinement on *F*^2^	Primary atom site location: structure-invariant direct methods
Least-squares matrix: full	Secondary atom site location: difference Fourier map
*R*[*F*^2^ > 2σ(*F*^2^)] = 0.033	Hydrogen site location: inferred from neighbouring sites
*wR*(*F*^2^) = 0.086	H atoms treated by a mixture of independent and constrained refinement
*S* = 1.02	*w* = 1/[σ^2^(*F*_o_^2^) + (0.0432*P*)^2^ + 0.3573*P*] where *P* = (*F*_o_^2^ + 2*F*_c_^2^)/3
2465 reflections	(Δ/σ)_max_ < 0.001
190 parameters	Δρ_max_ = 0.23 e Å^−^^3^
4 restraints	Δρ_min_ = −0.45 e Å^−^^3^

### Special details

**Table:**

Geometry. All e.s.d.'s (except the e.s.d. in the dihedral angle between two l.s. planes) are estimated using the full covariance matrix. The cell e.s.d.'s are taken into account individually in the estimation of e.s.d.'s in distances, angles and torsion angles; correlations between e.s.d.'s in cell parameters are only used when they are defined by crystal symmetry. An approximate (isotropic) treatment of cell e.s.d.'s is used for estimating e.s.d.'s involving l.s. planes.
Refinement. Refinement of *F*^2^ against ALL reflections. The weighted *R*-factor *wR* and goodness of fit *S* are based on *F*^2^, conventional *R*-factors *R* are based on *F*, with *F* set to zero for negative *F*^2^. The threshold expression of *F*^2^ > σ(*F*^2^) is used only for calculating *R*-factors(gt) *etc*. and is not relevant to the choice of reflections for refinement. *R*-factors based on *F*^2^ are statistically about twice as large as those based on *F*, and *R*-factors based on ALL data will be even larger.

### Fractional atomic coordinates and isotropic or equivalent isotropic displacement parameters (Å^2^)

**Table:**

	*x*	*y*	*z*	*U*_iso_*/*U*_eq_	
Zn1	0.0000	0.5000	0.5000	0.02474 (14)	
S1	0.01224 (8)	0.60147 (5)	0.17445 (2)	0.02955 (17)	
N2	0.1353 (3)	0.80094 (16)	0.20795 (6)	0.0297 (5)	
N1	0.0402 (3)	0.65524 (16)	0.26077 (6)	0.0264 (5)	
O4	0.2360 (2)	0.41441 (14)	0.47861 (5)	0.0314 (4)	
H4WB	0.307 (3)	0.396 (2)	0.4993 (7)	0.038*	
H4WA	0.302 (3)	0.450 (2)	0.4603 (7)	0.038*	
C1	0.0700 (3)	0.6981 (2)	0.21845 (8)	0.0256 (5)	
C10	0.0382 (3)	0.6859 (2)	0.12255 (8)	0.0283 (5)	
H10A	−0.0412	0.7522	0.1244	0.034*	
H10B	0.1658	0.7114	0.1198	0.034*	
O1	−0.0740 (3)	0.51902 (14)	0.08678 (6)	0.0396 (5)	
C5	0.0435 (3)	0.67551 (18)	0.34366 (8)	0.0236 (5)	
C4	0.0801 (3)	0.72292 (19)	0.29679 (7)	0.0241 (5)	
C11	−0.0136 (3)	0.6163 (2)	0.08050 (8)	0.0269 (5)	
N3	−0.0130 (2)	0.58138 (16)	0.43217 (6)	0.0255 (4)	
O2	0.0038 (2)	0.66402 (14)	0.04128 (6)	0.0339 (4)	
O3	−0.1746 (3)	0.36656 (14)	0.47517 (6)	0.0342 (4)	
H3WB	−0.118 (3)	0.3056 (17)	0.4696 (9)	0.041*	
H3WA	−0.269 (3)	0.355 (2)	0.4908 (8)	0.041*	
C7	0.0205 (3)	0.6920 (2)	0.42625 (8)	0.0287 (6)	
H7A	0.0259	0.7377	0.4525	0.034*	
C3	0.1516 (3)	0.83095 (19)	0.28983 (8)	0.0295 (5)	
H3B	0.1814	0.8781	0.3147	0.035*	
C9	0.0026 (3)	0.5610 (2)	0.34959 (8)	0.0301 (6)	
H9A	−0.0059	0.5135	0.3239	0.036*	
C8	−0.0249 (3)	0.5184 (2)	0.39326 (8)	0.0309 (6)	
H8A	−0.0534	0.4418	0.3962	0.037*	
C2	0.1763 (4)	0.86515 (19)	0.24447 (8)	0.0311 (6)	
H2B	0.2245	0.9372	0.2391	0.037*	
C6	0.0476 (3)	0.7421 (2)	0.38362 (8)	0.0294 (5)	
H6A	0.0685	0.8197	0.3815	0.035*	

### Atomic displacement parameters (Å^2^)

**Table:**

	*U*^11^	*U*^22^	*U*^33^	*U*^12^	*U*^13^	*U*^23^
Zn1	0.0297 (2)	0.0257 (2)	0.0188 (2)	0.00061 (16)	0.00071 (16)	−0.00053 (15)
S1	0.0392 (4)	0.0303 (3)	0.0191 (3)	−0.0049 (3)	−0.0001 (3)	0.0019 (2)
N2	0.0318 (11)	0.0314 (11)	0.0259 (10)	−0.0026 (9)	−0.0017 (9)	0.0039 (9)
N1	0.0317 (12)	0.0287 (11)	0.0187 (10)	0.0006 (8)	−0.0007 (8)	0.0016 (8)
O4	0.0326 (10)	0.0363 (10)	0.0255 (9)	0.0049 (8)	0.0041 (8)	0.0036 (8)
C1	0.0233 (12)	0.0312 (13)	0.0222 (12)	0.0020 (10)	−0.0017 (9)	−0.0006 (10)
C10	0.0328 (14)	0.0304 (13)	0.0216 (12)	−0.0034 (10)	0.0017 (10)	0.0032 (10)
O1	0.0562 (12)	0.0353 (10)	0.0274 (10)	−0.0098 (9)	−0.0089 (9)	0.0006 (8)
C5	0.0238 (12)	0.0261 (12)	0.0210 (12)	0.0020 (9)	−0.0029 (9)	0.0013 (10)
C4	0.0218 (12)	0.0287 (12)	0.0217 (12)	0.0019 (10)	−0.0018 (10)	0.0008 (10)
C11	0.0275 (13)	0.0307 (13)	0.0226 (12)	0.0033 (10)	0.0000 (10)	0.0015 (10)
N3	0.0291 (11)	0.0275 (11)	0.0200 (10)	0.0010 (8)	−0.0004 (8)	−0.0019 (8)
O2	0.0474 (11)	0.0340 (10)	0.0203 (9)	0.0031 (8)	0.0014 (7)	0.0017 (7)
O3	0.0373 (11)	0.0329 (10)	0.0325 (10)	−0.0045 (8)	−0.0010 (8)	−0.0016 (8)
C7	0.0347 (14)	0.0288 (13)	0.0225 (12)	0.0031 (10)	−0.0018 (10)	−0.0062 (10)
C3	0.0339 (14)	0.0271 (13)	0.0276 (13)	−0.0002 (10)	−0.0047 (11)	−0.0022 (10)
C9	0.0393 (15)	0.0289 (13)	0.0220 (12)	−0.0023 (11)	−0.0014 (10)	−0.0066 (10)
C8	0.0436 (16)	0.0257 (12)	0.0233 (13)	−0.0046 (11)	−0.0005 (11)	−0.0001 (10)
C2	0.0328 (14)	0.0267 (13)	0.0339 (14)	−0.0042 (11)	−0.0028 (11)	0.0045 (11)
C6	0.0358 (14)	0.0247 (12)	0.0277 (13)	0.0001 (10)	−0.0036 (11)	−0.0015 (10)

### Geometric parameters (Å, °)

**Table:**

Zn1—O4	2.070 (2)	C5—C9	1.393 (3)
Zn1—O4^i^	2.070 (2)	C5—C6	1.392 (3)
Zn1—O3^i^	2.137 (2)	C5—C4	1.483 (3)
Zn1—O3	2.137 (2)	C4—C3	1.388 (3)
Zn1—N3^i^	2.176 (2)	C11—O2	1.267 (3)
Zn1—N3	2.176 (2)	N3—C7	1.337 (3)
S1—C1	1.753 (3)	N3—C8	1.346 (3)
S1—C10	1.804 (3)	O3—H3WB	0.843 (16)
N2—C2	1.328 (3)	O3—H3WA	0.822 (17)
N2—C1	1.335 (3)	C7—C6	1.375 (3)
N1—C1	1.336 (3)	C7—H7A	0.9300
N1—C4	1.339 (3)	C3—C2	1.377 (3)
O4—H4WB	0.810 (16)	C3—H3B	0.9300
O4—H4WA	0.821 (16)	C9—C8	1.367 (4)
C10—C11	1.508 (3)	C9—H9A	0.9300
C10—H10A	0.9700	C8—H8A	0.9300
C10—H10B	0.9700	C2—H2B	0.9300
O1—C11	1.240 (3)	C6—H6A	0.9300
			
O4—Zn1—O4^i^	180.00 (8)	C6—C5—C4	122.3 (2)
O4—Zn1—O3^i^	88.60 (9)	N1—C4—C3	121.0 (2)
O4^i^—Zn1—O3^i^	91.40 (9)	N1—C4—C5	116.1 (2)
O4—Zn1—O3	91.40 (9)	C3—C4—C5	122.9 (2)
O4^i^—Zn1—O3	88.60 (9)	O1—C11—O2	125.1 (2)
O3^i^—Zn1—O3	180.0	O1—C11—C10	118.2 (2)
O4—Zn1—N3^i^	90.94 (7)	O2—C11—C10	116.6 (2)
O4^i^—Zn1—N3^i^	89.06 (7)	C7—N3—C8	116.3 (2)
O3^i^—Zn1—N3^i^	89.99 (8)	C7—N3—Zn1	122.44 (15)
O3—Zn1—N3^i^	90.01 (8)	C8—N3—Zn1	120.33 (17)
O4—Zn1—N3	89.06 (7)	Zn1—O3—H3WB	113.9 (19)
O4^i^—Zn1—N3	90.94 (7)	Zn1—O3—H3WA	114.9 (19)
O3^i^—Zn1—N3	90.01 (8)	H3WB—O3—H3WA	111 (3)
O3—Zn1—N3	89.99 (8)	N3—C7—C6	123.9 (2)
N3^i^—Zn1—N3	180.0	N3—C7—H7A	118.0
C1—S1—C10	102.37 (13)	C6—C7—H7A	118.0
C2—N2—C1	114.66 (19)	C2—C3—C4	116.9 (2)
C1—N1—C4	116.4 (2)	C2—C3—H3B	121.5
Zn1—O4—H4WB	115.2 (19)	C4—C3—H3B	121.5
Zn1—O4—H4WA	114.8 (18)	C8—C9—C5	119.9 (2)
H4WB—O4—H4WA	104 (3)	C8—C9—H9A	120.0
N2—C1—N1	127.3 (2)	C5—C9—H9A	120.0
N2—C1—S1	120.71 (17)	N3—C8—C9	123.5 (2)
N1—C1—S1	111.96 (18)	N3—C8—H8A	118.2
C11—C10—S1	109.75 (17)	C9—C8—H8A	118.2
C11—C10—H10A	109.7	N2—C2—C3	123.6 (2)
S1—C10—H10A	109.7	N2—C2—H2B	118.2
C11—C10—H10B	109.7	C3—C2—H2B	118.2
S1—C10—H10B	109.7	C7—C6—C5	119.4 (2)
H10A—C10—H10B	108.2	C7—C6—H6A	120.3
C9—C5—C6	116.8 (2)	C5—C6—H6A	120.3
C9—C5—C4	121.0 (2)		

Symmetry codes: (i) −*x*, −*y*+1, −*z*+1.

### Hydrogen-bond geometry (Å, °)

**Table:**

*D*—H···*A*	*D*—H	H···*A*	*D*···*A*	*D*—H···*A*
O4—H4WB···O2^ii^	0.81 (2)	1.96 (2)	2.759 (3)	170 (3)
O4—H4WA···O1^iii^	0.82 (2)	1.82 (2)	2.632 (3)	172 (2)
O3—H3WB···O2^iv^	0.84 (2)	1.89 (2)	2.728 (3)	176 (3)
O3—H3WA···O2^v^	0.82 (2)	2.24 (2)	3.060 (3)	172 (3)

Symmetry codes: (ii) −*x*+1/2, −*y*+1, *z*+1/2; (iii) *x*+1/2, *y*, −*z*+1/2; (iv) −*x*, *y*−1/2, −*z*+1/2; (v) −*x*−1/2, −*y*+1, *z*+1/2.
